# On Suppression of Urine after Parturition

**Published:** 1810-11

**Authors:** Sam. Merriman

**Affiliations:** Curzon-street, May Fair


*To the Editors of the Medical and Physical Journal.*

Gentlemen,

If you think the following Case, first of suppression, and afterwards of incontinence of urine, after parturition, deserving of publication, I will thank you to cause it to be inserted in the Medical and Physical Journal.

I remain, &c.

*Curzon-street, May Fair,* SAM. MERRIMAN.

*Oct.* 11, 1810.

Mary Chapman, 21 years of age, a patient of the Westminster General Dispensary, residing in the neighbourhood of Berwick-street, Soho, was taken in labour of her first child on the 13th October, 1809. Her waters *broke* on the preceding day, but the pains of labor did not come on till about 12 o'clock at noon of the 13th. From this time they continued frequent, and violent for twelve hours, when the strength of the poor woman, and the patience of her friends being exhausted, they requested that the midwife should send for further assistance, and I was accordingly called up to her, between one and two o'clock in the morning of the 14th.

When I arrived at the house, the midwife informed me, that the child's head had fallen so low into the pelvis, that the vertex was actually protruding through the *os externum*, and that she expected it to be born in a few more pains: she acknowledged, however, that the pains were become less efficacious during the last two hours, and that the labour had not advanced since; and she conjectured with much appearance of reason, that this diminution of uterine action was occasioned by the bladder being full of urine, as the patient had made no water during the whole continuance of the labor. On hearing this report, I laid my hand upon the abdomen, and clearly distinguished by the feel, that the bladder was very full; I concluded therefore, that there would be nothing more to do than to introduce the catheter, and empty the bladder, after which it was to be expected that the pains would recover their former strength, and that the delivery would be accomplished without any further difficulty.

The patient being therefore placed in a convenient posture, I proceeded to introduce the catheter; in doing which, I found that the child's head was as the midwife had stated, so low in the pelvis, as to render it almost certain, that a few pains would expel it; but on passing my finger into the vagina, it [the vagina] felt so excessively hot and burning, as convinced me that the delivery ought not to be trusted to the efforts of nature, but that the child must be removed with all proper expedition, as otherwise there was great reason to apprehend that mortification and sloughing of the vagina would ensue. It was however first necessary to draw off the water; I therefore introduced the catheter as gently as possible, (notwithstanding which, it gave her very great pain, from the inflamed and irritable state of the *meatus urinarius)* and drew off nearly a quart of very high-coloured urine. In a short time afterwards I introduced the forceps, and delivered the woman of a female child, which was restored to life with considerable difficulty. In rather less than twenty minutes the placenta was expelled, and a profuse discharge from the uterus took place at the same time. She now had an opiate given to her, and I left her with very strict injunctions respecting her diet and management.

Before twelve hours had expired, I received information that this patient was suffering great pain from a constant urgency to make water and an inability of voiding it. I therefore again had recourse to the catheter, and emptied the bladder. The orifice of the *meatus urinarius* was swollen and tender, and the passing of the instrument occasioned her much pain; but the sense of heat and burning in the vagina was much diminished.

The usual emollient, aperient and anodyne remedies were employed to relieve her complaint; but it was necessary to have constant recourse to the catheter for eight or nine days, at at the end of which time she began to pass her urine naturally; but she now entirely lost the power of retaining it, and suffered the various inconveniences which accompany an incontinence of urine. I began therefore to fear that an ulceration had taken place, and had made a communication between the bladder and vagina; but after a very accurate examination of the parts on two different occasions, I was fully satisfied that no such accident had happened. During the course of ten or twelve days this continual discharge of urine continued, notwithstanding the use of a variety of remedies that were employed to counteract it; at length I determined to try the effect of the uva ursi, and accordingly prescribed a scruple of the powder to be taken three times a day. After a few days, Mrs. Chapman thought she derived benefit from this medicine, and was therefore very willing to persevere in its use: it was continued for a fortnight longer, by which time the incontinence of urine was removed, and her health and strength perfectly restored.

It has been laid down as a rule in practice, and it is one of those rules, which, being founded in reason and experience, ought not lightly to be deviated from, that the head of the child should be in a situation capable of being delivered by the forceps, for at least *six hours* before they are had recourse to. As a means of guarding against a rash and unnecessary use of instruments in the practice of midwifery, this rule is highly commendable; but a rigid observance of it, in the case I have related, would have proved extremely injurious, if not fatal to the patient, as it is apparent that a very high degree of inflammation in the vagina, and parts adjacent, was just upon the point of taking place: and had the head of the child been suffered to remain there much longer, so much inflammation must necessarily have come on, as might have proved uncontroulable. It may be said, that when the bladder was evacuated, the pains would have returned; but it is possible that the return of uterine action might not have taken place for many hours, and in the mean time incalculable mischief would have been going on; upon the whole therefore, I am persuaded that this was one of the cases in which it was prudent to break through a useful rule in practice.

A relaxation, or want of contractile power in the *sphincter vesicæ*, is sometimes observed after a suppression of urine, arising probably from the frequent introduction of the catheter, and consequent dilatation of the parts; but the incontinence of urine rarely proceeds to so great a degree as in this patient, nor does it last so long. I was led to the use of the uva ursi from the celebrity it formerly attained as a remedy medy in the disorders of the urinary organs in the practice of *de Haen*, and other German physicians. A blister over the sacrum would perhaps have been a more speedy remedy, but the constant discharge of urine had produced so much cutaneous inflammation all over the lower parts of the body, as to render the application of a blister unadvisable, at least in the first instance; and as the woman was daily mending under the use of the uva ursi, I was afterwards unwilling to discontinue it.

The uva ursi, as a remedy in disorders of these organs, is almost entirely fallen into disuse. It has experienced the fate of many once celebrated and fashionable articles of the Materia Medica; having at first been too highly and unjustly extolled, it has now perhaps sunk into unmerited oblivion. Lewis, in his "Experimental History of the Materia Medica," says of it, "In all cases that have come to my "knowledge, it produced great sickness, and uneasiness, "without any apparent benefit, though continued for a "month." It is probable that in these cases the uva ursi was given in too large doses, for I observed no such ill effects in the doses I prescribed for Mrs. Chapman.

